# Automated 3D Cobb Angle Measurement Using U-Net in CT Images of Preoperative Scoliosis Patients

**DOI:** 10.1007/s10278-024-01211-w

**Published:** 2024-08-08

**Authors:** Lening Li, Teng Zhang, Fan Lin, Yuting Li, Man-Sang Wong

**Affiliations:** 1 Department of Biomedical Engineering, The Hong Kong Polytechnic University, Hong Kong, China; 2 School of Artificial Intelligence, Nanjing University of Information Science and Technology, Nanjing, China; 3 Institute for Artificial Intelligence in Medicine, Nanjing University of Information Science and Technology, Nanjing, China; 4 Department of Radiology, The First Affiliated Hospital of Shenzhen University, Shenzhen, China; 5 Health Science Center, Shenzhen Second People’s Hospital, Shenzhen, China; 6 Nurturing Center of Jiangsu Province for State Laboratory of AI Imaging & Interventional Radiology, Department of Radiology, Zhongda Hospital, Medical School of Southeast University, Nanjing, China

**Keywords:** Scoliosis, Vertebra segmentation, Cobb angle, U-net, NURBS-net

## Abstract

To propose a deep learning framework “SpineCurve-net” for automated measuring the 3D Cobb angles from computed tomography (CT) images of presurgical scoliosis patients. A total of 116 scoliosis patients were analyzed, divided into a training set of 89 patients (average age 32.4 ± 24.5 years) and a validation set of 27 patients (average age 17.3 ± 5.8 years). Vertebral identification and curve fitting were achieved through U-net and NURBS-net and resulted in a Non-Uniform Rational B-Spline (NURBS) curve of the spine. The 3D Cobb angles were measured in two ways: the predicted 3D Cobb angle (PRED-3D-CA), which is the maximum value in the smoothed angle map derived from the NURBS curve, and the 2D mapping Cobb angle (MAP-2D-CA), which is the maximal angle formed by the tangent vectors along the projected 2D spinal curve. The model segmented spinal masks effectively, capturing easily missed vertebral bodies. Spoke kernel filtering distinguished vertebral regions, centralizing spinal curves. The SpineCurve Network method’s Cobb angle (PRED-3D-CA and MAP-2D-CA) measurements correlated strongly with the surgeons’ annotated Cobb angle (ground truth, GT) based on 2D radiographs, revealing high Pearson correlation coefficients of 0.983 and 0.934, respectively. This paper proposed an automated technique for calculating the 3D Cobb angle in preoperative scoliosis patients, yielding results that are highly correlated with traditional 2D Cobb angle measurements. Given its capacity to accurately represent the three-dimensional nature of spinal deformities, this method shows potential in aiding physicians to develop more precise surgical strategies in upcoming cases.

## Introduction

Idiopathic scoliosis is a complex, three-dimensional spinal deformity typically recognized by the Cobb angle (CA) measured from 2D radiographs [1]. Severe cases of spinal curvature necessitate surgical intervention [2]. The significance of CA is well documented, proving its value in selecting fusion strategies, forecasting postoperative curve corrections [3], and evaluating spontaneous curve correction in specific patient groups [4]. Furthermore, CA aids in identifying factors that influence postoperative complications [5] and enhances the accuracy of predictive models for surgical outcomes [6, 7]. It plays a significant role in spinal height correction, associated with substantial height gains post-surgery [6–8], and affects crucial surgical considerations like procedure duration, blood loss, and the need for blood transfusions [9–11]. It also predicts the risk of “adding-on” phenomena postoperatively [12] and the success rates of selective thoracic fusions [13]. The preoperative CA magnitude is also connected to surgical challenges and risk evaluations [14, 15], underscoring its widespread impact on scoliosis treatment and postoperative improvements, including self-image enhancements [16]. 

Considering the spine’s intricate anatomy and the inherently three-dimensional nature of deformity, CA measurement based on computed tomography (CT) scans could yield more comprehensive information [17, 18]. While numerous studies and public databases have implemented vertebral segmentation based on CT, further advancements in CA measurements are seldom discussed. Previous CT-based CA calculations primarily focused on mild and moderate scoliosis cases [19–21]. It is undeniable that CT imaging is typically acquired for presurgical cases suffering from the severe spine curve to avoid unnecessary ionizing radiation exposure, but curve estimation in this group is largely understudied. 

This study aims to address these gaps by proposing an SpineCurve-net for automatic CA measurement in CT images of presurgical severe scoliosis patients. This method employs the U-net and NURBS-net architecture for the segmentation of vertebrae and subsequently calculates the Cobb angle. Statistical analyses were conducted to compare the 3D CA measured by SpineCurve-net to the traditional CA measured in 2D radiography. This focus addresses the unique challenges and complexities associated with severe spinal deformities, providing critical insights for preoperative planning.

## Materials and Methods

### Subjects and Image Acquisition

In this retrospective study, subjects were selected based on the following inclusion criteria: diagnosis of idiopathic scoliosis (IS), congenital scoliosis, or degenerative scoliosis; age within the range as follows: adolescent idiopathic scoliosis: 10 to 16 years, congenital scoliosis: 0 to 10 years, and degenerative scoliosis: 55 to 85 years; coronal Cobb angle between 10° and 85°; and with preoperative CT images and posteroanterior (PA) X-ray images of the entire spine. The exclusion criteria included any prior surgical interventions of the spine or other underlying conditions that might influence the spinal profile.

This study includes a range of scoliosis severities (mild, moderate, and severe) to develop a robust model. However, the primary emphasis is on severe presurgical cases to enhance the model’s applicability in complex clinical scenarios. This approach ensures that the model is well-equipped to handle varying degrees of spinal deformity, improving its generalizability and clinical relevance. A total of 116 patients with scoliosis were included in the analysis, among which 89 were allocated to the training set, while the remaining 27 were designated for testing. Records from The First Affiliated Hospital of Shenzhen University were examined retrospectively to identify suitable scoliosis patients who underwent selective spinal fusion between the years 2016 and 2022. Ethical approval was obtained from the author’s Institutional Review Board before the commencement of the study (Ethical Approval No. 20220920004).

All the recruited subjects were imaged pre-operatively in the supine position with a CT scanner (Siemens, with parameters set at 120 kV, mAS 212, slice 5 mm, pitch 0.8, thickness 1.5 mm).

### Vertebra Segmentation

#### Manual Segmentation

All CT images were resampled to 0.90 × 0.35 × 0.35 mm^3, on which the vertebral columns were segmented manually by a skilled radiologist (LL) using the open-source software ITK-SNAP (University of Pennsylvania and University of North Carolina at Chapel Hill, USA), and were subsequently reviewed by another radiologist (FL). The spinal columns were labeled without distinguishing between individual vertebrae. After this, the images were arbitrarily divided into a training dataset (*n* = 80) and a testing dataset (*n* = 10).

#### Deep Learning-Based Segmentation

A 3D U-net was trained by using the training dataset via the U-Net method (https://github.com/MIC-DKFZ/nnUNet). Image patches of 112 × 160 × 128 pixels were introduced to the U-net, and the batch size was designated to 2. Five downsampling procedures were executed to produce a feature map with dimensions of 7 × 5 × 4. The initial convolution channel count stood at 32 and increased twofold after each downsampling procedure. The Leaky Rectified Linear Unit (ReLU) served as the activation function. Throughout the training phase, both the dice coefficient and cross-entropy metrics were reduced through stochastic gradient descent at a learning rate of 0.01. The entire training phase spanned 1000 epochs.

The 3D nature of the U-net architecture inherently considers the correlation between slices, ensuring that the spatial relationships between adjacent slices are preserved. This approach allows for a more accurate and coherent segmentation of the vertebral bodies across the entire volume of CT images.

### Spinal Curve Fitting

#### Removal of Spinous and Transverse Processes

The U-net segmented whole vertebra regions, containing vertebra body, transverse process, and spinous process. Due to the significant structural alterations in patients with scoliosis, the transverse process and spinous process could introduce biases to estimated spinal curves, and thus were removed by a technique called spoke kernel filtering.

**Fig. 1:**
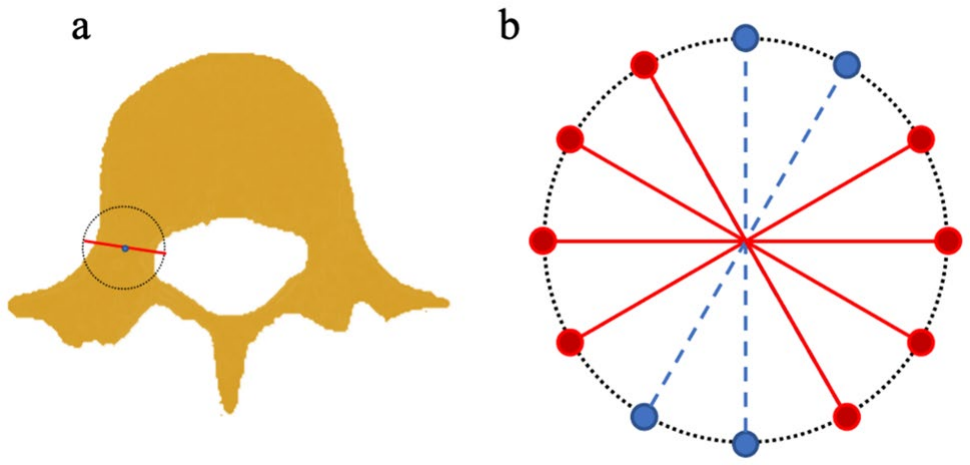
Spoke kernel filtering. **a** Center of the spoke kernel was applied to each pixel of spine segmentation. **b** For each diameter, if both end points were located outside the spine region (red points), its traversing pixels would be removed from the segmentation mask. Otherwise, the traversing points would be preserved (blue dashed lines)

The spoke kernel filtering could exclude transverse and spinous processes, as well as separating connected vertebra regions caused by serious scoliosis. As illustrated in Fig. 1, the spoke kernel aims to eradicate narrow “bottleneck” regions. Utilizing the spoke kernel, neighboring vertebrae, in conjunction with transverse and spinous processes, can be adequately differentiated.

The spoke kernel comprises a circular mask, devised through the midpoint circle algorithm. Line segments spanning the circular epicenter are formulated through the midpoint line algorithm, and they function to bridge centrosymmetric circular coordinates. In spoke kernel filtering, the circle center traverses all spinal pixels in each segmentation mask of vertebral colume. If both ends of a line segment are located on non-spinal structures, all points on this line are considered as candicates to be excluded from the segmentation mask. These line coordinates are collectively discarded upon the finalization of the scan of all spinal voxels. The spoke kernel filtering can be regarded as a speical version of morphological openning operation that removes narrow bridges connecting large regions. Although some boundary voxels could be removed after spoke kernel filtering, the majority remains and vertebral shapes are smoothed. The spoke kernel radius was initially set at 8 mm and adjusted manually to accommodate variances in image dimensions. Therefore, after spoke kernel filtering, detached minuscule regions are removed, utilizing a threshold of 500 voxel count.

#### NURBS Fitting for Ground Truth

After Spoke Kernel Filtering, vertebral domains were clearly segmented.

For every vertebra body, K-means clustering was executed to aggregate voxels into clusters based on their spatial localization. The quantity of clusters was designated as 1/1000 of the total number of voxels. This clustering method utilized the 3D coordinates of each voxel to group them into clusters, ensuring that the spatial distribution of voxels within each vertebra body was accurately represented. The primary feature used for clustering was the location of the voxels in the 3D space, which allowed for an effective aggregation of voxels into anatomically coherent clusters. Three-dimensional Non-Uniform Rational B-Spline (NURBS) curves were fitted to the cluster centroids using the least squares approach. Cubic B-spline basis functions (degree = 3) were harnessed, and the number of control points was provisionally fixed at 8. The resulting 3D curve is referred to as ground truth in following deep learning framework.

#### NURBS-net Curve Fitting

**Fig. 2:**
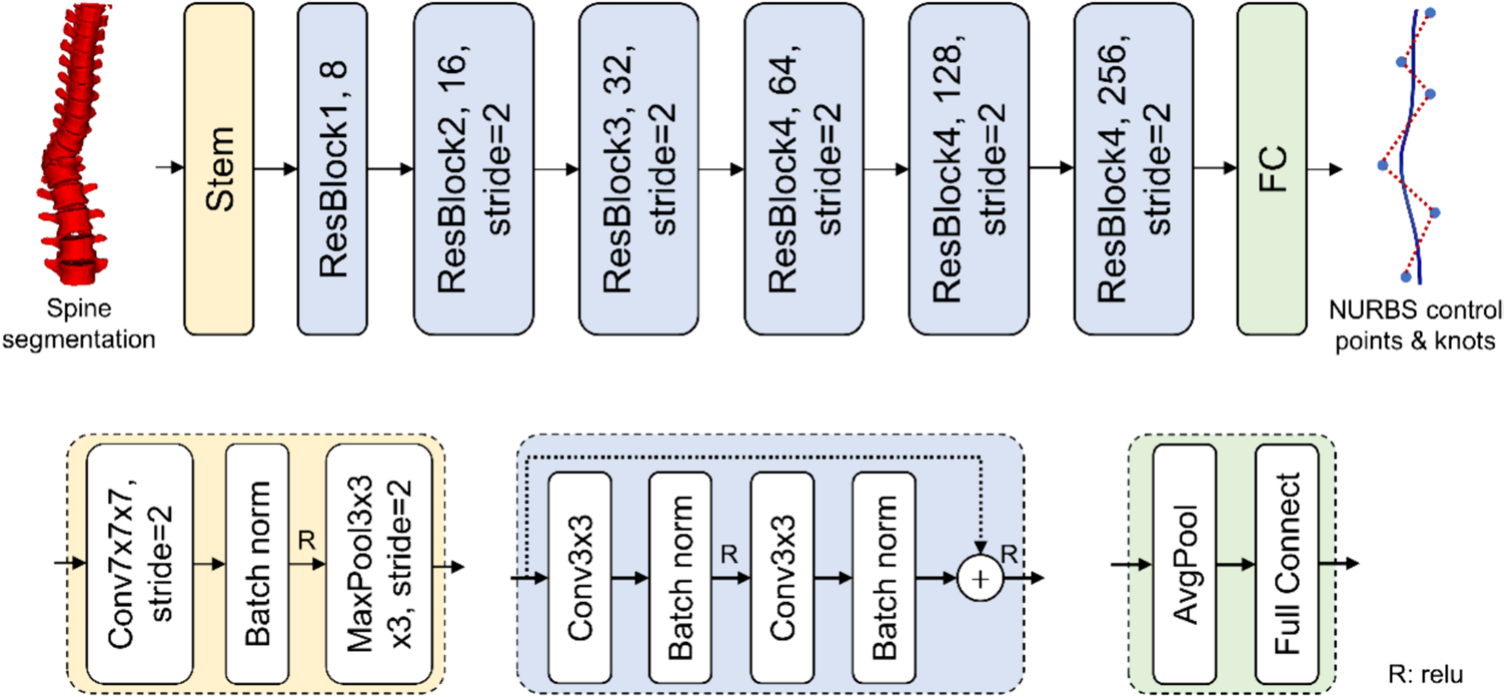
Deep learning network for spinal curve fitting. This network employed Res-Net as the backbone. Spine segmentation was used as input, and control points and knots of Non-Uniform Rational B-Spline (NURBS) curves were used as the output

The NURBS-net was subsequently applied to predict NURBS control points and knot vectors from spinal segmentation outcomes of UNet. The NURBS-net adopted ResNet as its foundational structure (Fig. 2) and was cultivated using the analogous training dataset as that of UNet. Throughout the training endeavor, spine segmentation and “Ground-truth curve” underwent random affine transformations for data enrichment. Given that the initial and terminal quartet of knot vector values invariably stand at 0 and 1, only the quartet of intermediate knot values and 8 control points are projected by the curve-net. The loss function was evaluated as the following equation:

1 $$loss=\frac{{\Vert {ctrlpts}_{GT}-{ctrlpts}_{pred}\Vert }_{L2}^{2}}{{\Vert {ctrlpts}_{GT}\Vert }_{L2}^{2}}+\frac{{\Vert {knots}_{GT}-{knots}_{pred}\Vert }_{L2}^{2}}{{\Vert {knots}_{GT}\Vert }_{L2}^{2}},$$

where $ctrlpts$ and $knots$ indicate control points and knot values, $GT$ and $pred$ indicate ground truth and predicted values, respectively. The learning rate was set to 0.001, and a total of 1000 epochs was taken by the training process. Symmetric mean of minimum distance was used to evaluate the distance between ground truth and deep learning-based curves.

### Measurement of 3D Cobb Angle

Based on the predicted NURBS control points and knot vectors, NURBS-net-based curves, which is referred to as “NURBS-curve” in follow, with 100 points and corresponding tangent vectors were reconstructed for each patient. Pairwise angles between tangent vectors were calculated between the 5th to 95th points since curve tails could be affected by incomplete vertebral structure on the top and bottom of CT images. These measurements are performed on the entire volumetric image, allowing for a comprehensive analysis of the three-dimensional structure of the spine. For each point pair *p* and *q*, angle of their tangent vector *T*_*p*_ and *T*_*q*_ was calculated as arccosine of their dot product:

2 $$Angle\left(p,q\right)=\mathrm{arccos}({T}_{p}\cdot {T}_{q})$$

**Fig. 3:**
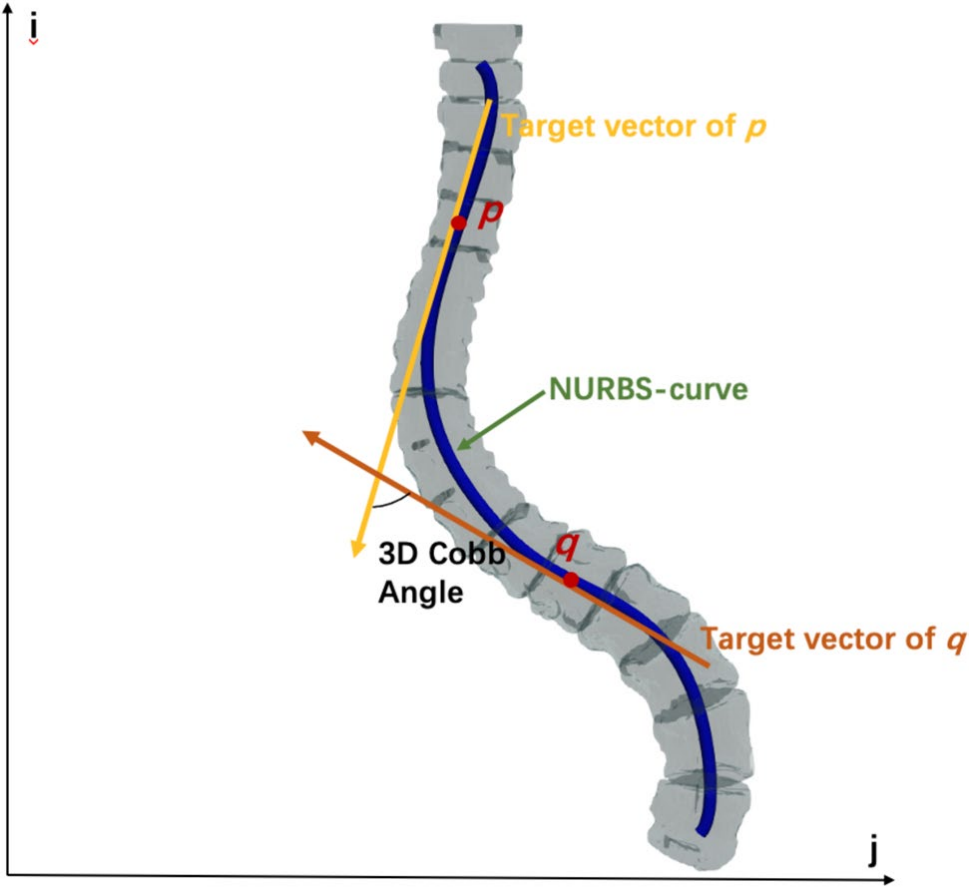
Illustration of the PRED-3D-CA-calculated method

A two-dimensional “angle map” was then built whose pixel intensity at *i*th row and *j*th colum represented the angle between *i*th and *j*th curve points. Then, this angle map was convoluted with a Gaussian smoothing kernel, in order to increase robustness of the measured angles and then define the predicted 3D Cobb angle (“PRED-3D-CA” in short) as the maximum value in the smoothed angle map from the NURBS-curve. Figure 3 illustrates the PRED-3D-CA calculation method. In addition, the NURBS-curve was projected onto the coronal plane, and the maximal angle among all the angles formed by the tangent vectors along the projected 2D spinal curve was calculated. This angle is named “MAP-2D-CA.”

### Statistics

Statistical evaluations were performed using SPSS (version 21, IBM, Chicago, IL, USA), maintaining a critical alpha threshold of 0.05. As 2D radiographs provide a projection of spinal curvature, 3D CT scans capture the full spatial deformity. We evaluated the consistency of 3D measurements with established 2D methods to ensure reliability. The traditional 2D Cobb angle, which surgeon annotated based on 2D radiographs, is referred to as “XRAY-CA.”

CT images were acquired with patients in the supine position, which may reduce the gravitational impact on spinal curvature compared to standing radiographs. To address this, a comparative analysis between supine CT and standing X-ray measurements are needed. The Pearson correlation coefficient was used to explore the relationships between SpineCurve-net based CA measurements (i.e., PRED-3D-CA and MAP-2D-CA) and the surgeons’ annotation (XRAY-CA). In addition, the mean absolute error (MAE), standard deviation (SD) and Bland–Altman’s plot were calculated to assess the difference between the XRAY-CA and the deep learning-based measurements (i.e., PRED-3D-CA and MAP-2D-CA).

## Results

A total of 116 patients with scoliosis were included in the analysis. In order to get more training dataset, 35 degenerative scoliosis were collected in the training set. The idiopathic scoliosis patients were randomly seperated into training and testing set. Therefore, the training set comprised 89 patients with an average age of 32.4 ± 24.5 years, while the validation set consisted of 27 patients who had surgery with an average age of 17.3 ± 5.8 years. Table 1 delineates the summary of the datasets used for both training and testing purposes.

**Table 1:** Summary of the data used in this study

Property	Database	
	Training	Testing
No. of patients	89	27
Adolescent Idiopathic Scoliosis (10–16 years)	54	27
Congenital scoliosis (0–10 years)	0	0
Degenerative scoliosis (55–85 years)	35	0
Gender, male/female	30/59	4/23
Age, mean ± SD (years)	32.4 ± 24.5	17.3 ± 5.8
No. of patients with spinal fusion	36	27

The dice coefficients between radiologist’s delineated ground truth segmentation and automatically generated spinal mask were measured in the training and testing datasets seperately. The accuracy, F1-score, precision, and recall were 0.996 ± 0.002, 0.945 ± 0.026, 0.920 ± 0.047, and 0.973 ± 0.014 respectively in the training dataset, and 0.992 ± 0.062, 0.877 ± 0.062, 0.818 ± 0.099, and 0.954 ± 0.045 in the testing dataset.

**Fig. 4:**
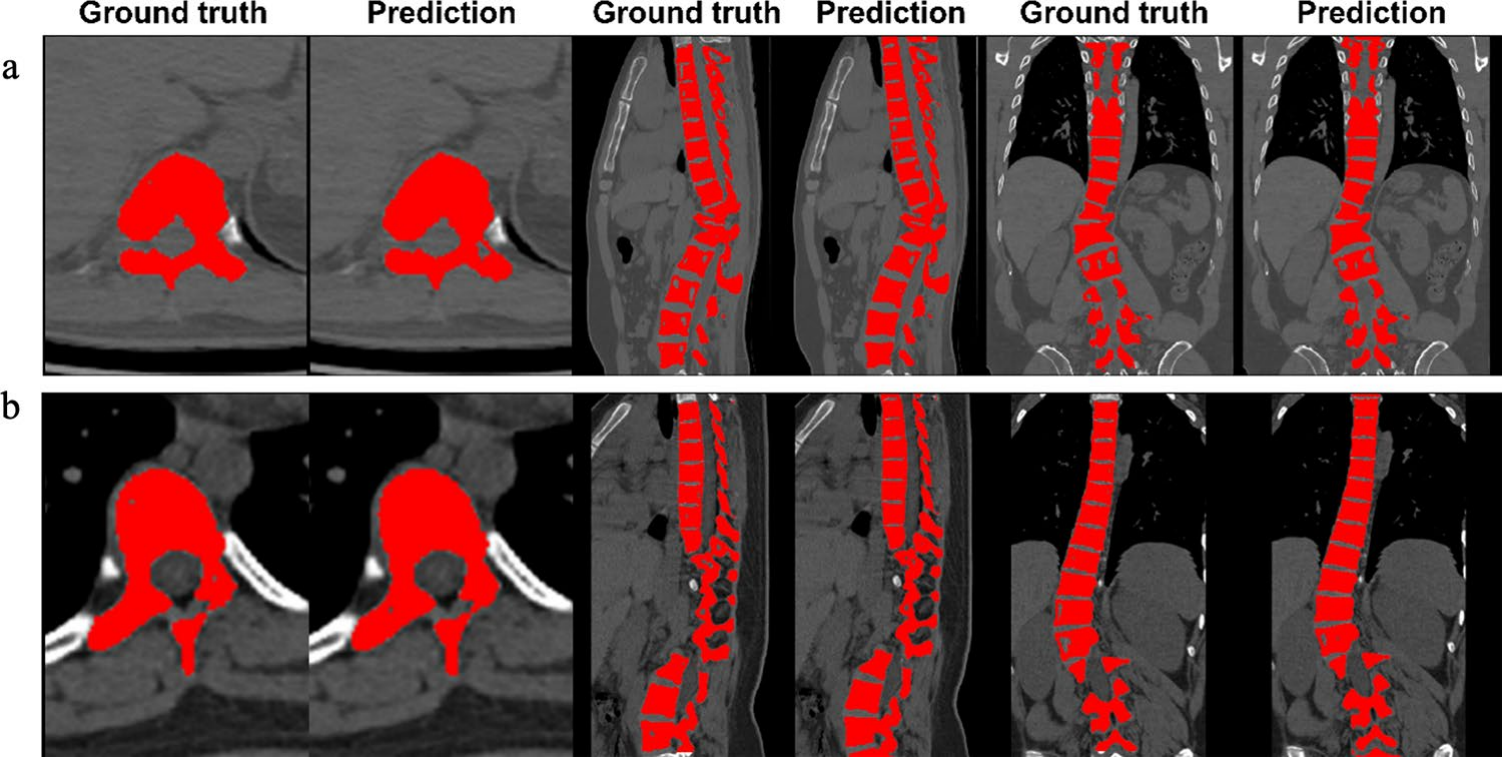
Spine segmentation results from two representative patients. **a** and **b** are results of a representative patient from the training and testing datasets, respectively. The images were cropped to remove irrelevant background regions

**Fig. 5:**
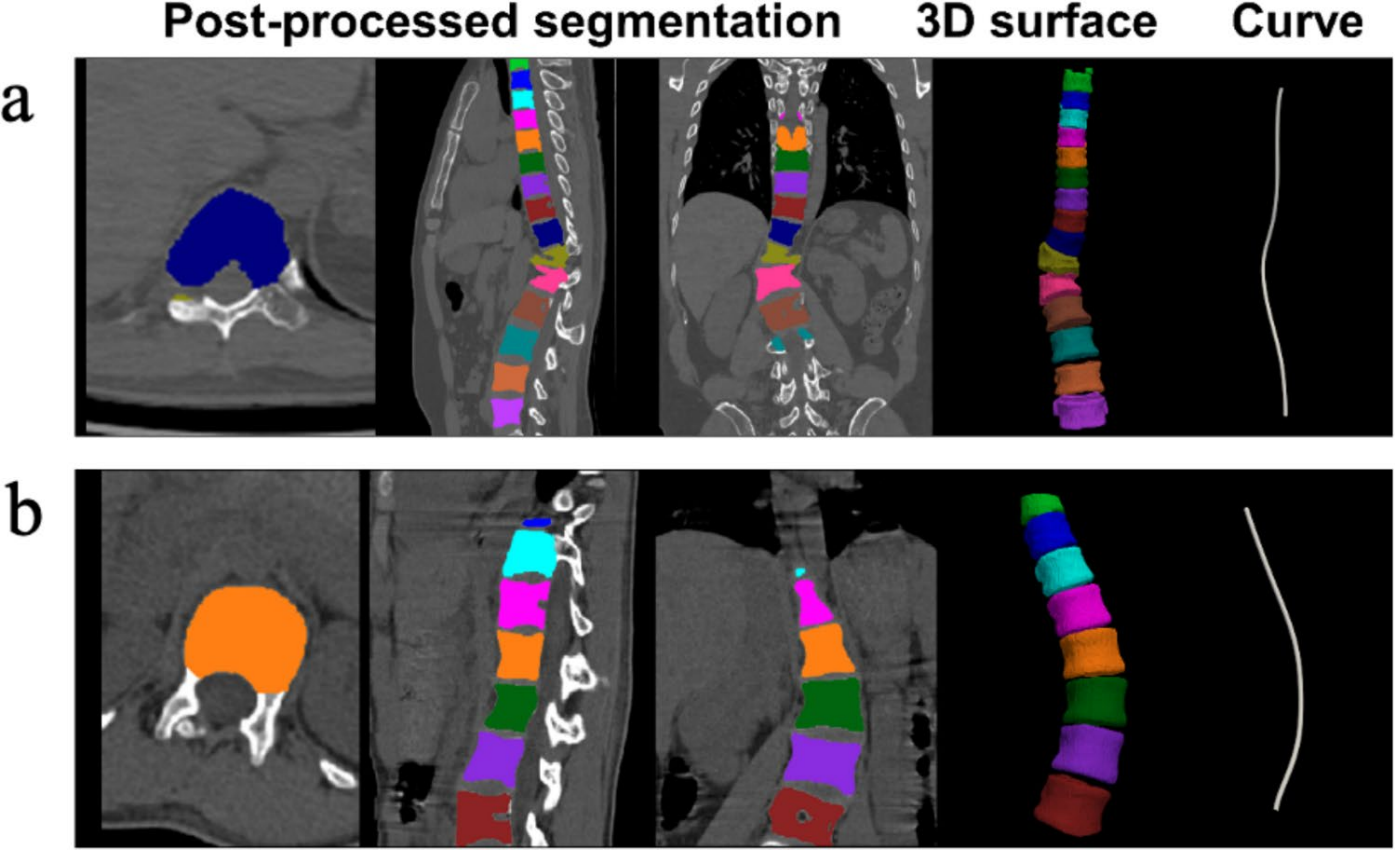
Post-processing and curve fitting. **a** and **b** are post-processing results of two representative patients from the training dataset. The curves shown in the last column are morphology-based curves

**Fig. 6:**
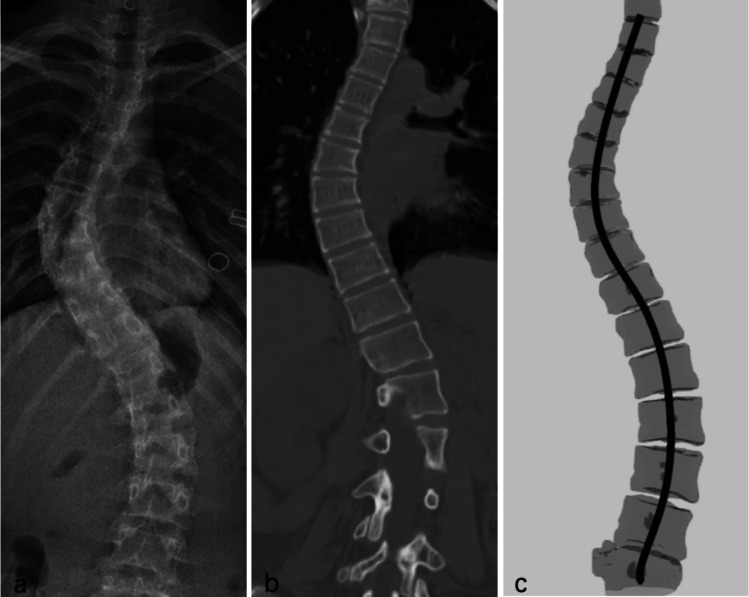
**a** The X-ray, **b** CT, and **c** NURBS-curve of one patient from the testing dataset

Visual evaluation confirms that the segmentation from the Unet model matched the ground truth well (e.g., Fig. 4). Notably, the Unet performs segmentation of vertebral bodies completely, including the top and bottom parts, which even outperformed the manual ground truth. For most patients (78/89), spoke kernel filtering with a radius of 8 mm achieved good performance. A smaller radius (6 mm) was applied to 7 patients, while larger ones (radius = 10 or 12 mm) were applied to 4 patients. The radius size was determined interactively based on the filtering results. Based on post-processing results, the NURBS-curves were reconstructed by NURBS-net and were located at the center of spinal regions (Fig. 5). The average curve distances between the Ground-truth-curve and NURBS-curve were 2.81 ± 1.09 mm and 3.65 ± 1.45 mm in the training and testing datasets, respectively. Figure 6 illustrates the X-ray image, CT image, and the NURBS-curve of one patient from the testing dataset.

In addition to comparing with the ground truth, comparisons with traditional 2D Cobb angle measurements were also performed.

Table 2 illstrates the comparison of XRAY-CA measurements against SpineCurve-net methods for estimating spinal curvature, i.e., PRED-3D-CA and MAP-2D-CA. The mean Cobb angle measured directly was 56.8° ± 12.8°, indicating a significant variability in spinal curvature across the sampled population. When compared to the XRAY-CA, PRED-3D-CA exhibited a closely aligned mean Cobb angle of 55.7° ± 13.8°, whereas MAP-2D-CA demonstrated a similar mean of 56.7° ± 13.8°, suggesting that both computational approaches had good agreements with the XRAY-CA. The Pearson correlation coefficient is 0.983 (*p* < 0.05) for PRED-3D-CA and 0.934 (*p* < 0.05) for MAP-2D-CA, relative to XRAY-CA. The mean absolute error (MAE) provides additional insight into the agreement with XRAY-CA, with PRED-3D-CA demonstrating a lower MAE of 2.4 ± 2.6 (°) compared to MAP-2D-CA’s MAE of 4.1 ± 4.9 (°). The mean absolute errors were 2.4° and 4.1°, respectively, indicating close alignment between the methods.

**Table 2:** Comparison of XRAY-CA measurements against SpineCuve Network methods

**Data category**	**XRAY-CA**	**PRED-3D-CA**	**MAP-2D-CA**
Mean Cobb (mean ± SD)	56.8° ± 12.8°	55.7° ± 13.8°	56.7° ± 13.8°
Pearson csorrelation coefficient	-	0.983 (*p* < 0.05)	0.934 (*p* < 0.05)
MAE ± SD	-	2.4 ± 2.6	4.1 ± 4.9

*SD* standard deviation, *MAE* mean absolute error

**Fig. 7:**
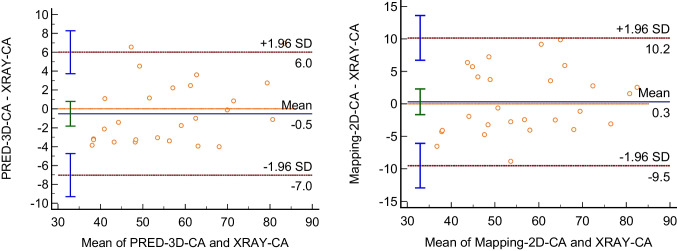
The Bland–Altman plot assessing the difference of the Cobb angle measurements, i.e., PRED-3D-CA, MAP-2D-CA, and XRAY-CA

Figure 7 gives a visual interpretation of the agreement between XRAY-CA and the computational results. For XRAY-CA versus PRED-3D-CA, the mean difference is 1.1°, with limits of agreement ranging from −4.0° to 6.2°. This narrow range indicates a strong agreement between XRAY-CA and PRED-3D-CA measurements. Conversely, the plot for XRAY-CA versus MAP-2D-CA shows a mean difference of 0.2°, with a broader limits of agreement from −9.5 to 9.8, indicating greater variability in the accuracy of MAP-2D-CA relative to XRAY-CA.

### Discussion

This study introduced an automated method for assessing the 3D Cobb angle in idiopathic scoliosis patients based on CT images. Utilizing a framework based on U-net, the method successfully segments the vertebral bodies and fits the spinal curve with another deep learning model, which was named NURBS-net. Based on the NURBS-curve, two types of Cobb angles were calculated. The feasibility of this approach was confirmed through its application to preoperative CT images from 27 patients who underwentspinal fusion surgery. Within spine segmentation, U-net algorithms exhibited pronounced accuracy in both training (0.996 ± 0.002) and testing (0.992 ± 0.062) datasets. The results surpass previous methods [20, 22, 23], particularly in the segmentation of moderate to severe spinal deformity [24].

As a post-processing step to prepare the segmentation results for spinal curve fitting, Spoke kernel filtering was proposed to isolate the vertebral body from other connected bony structures. Spoke kernel filtering discriminates between vertebral bodies and neighboring structures such as lamina, facet, and spinous process by verifying if two distant pixels, co-linear with a pixel, fall within the segmentation boundary. This step ensures that the adapted curve conforms closely to the vertebral body, countering influences of other spinal sections. Subsequent processing via NURBS curve fitting resulted in smooth curve of the spinal trajectories. The outlined protocol for spinal curve assessment integrates morphological analyses and NURBS curve fitting. NURBS curve fitting is based on the clusters generated from K-means clustering on the segmentation mask to alleviate computational burdens. NURBS trajectories were determined by a limited set of control points and knots, streamlining a smooth trajectory, which blurred out minor deviations in segmentation and subsequent processing.

Previous methods for 3D Cobb angle calculation can be largely grouped into two types, namely “endplate-based” and “curve-based” [25]. Endplate-based methods involve calculating the largest angle between the different endplates based on the 3D segmentation of the vertebral bodies. As mentioned in [20], a set of points is extracted along the edges, using anterior and posterior corners as reference points. Lines are then fitted to these points using the least squares method, with the angle between these fitted lines representing the 3D Cobb angle. However, the accuracy of this method is limited by the precision with which points can be detected. Another endplate-based method, detailed in [21], used the marching cubes technique to approximate the spinal surface. Seed points were manually selected, and a region-growing method is employed to determine the upper and lower endplates. This method requires manual intervention to locate seed points and relies on the accuracy of segmentation and the marching cubes algorithm. In contrast, the curve-based methods generate a spinal curve based on the segmentation of the vertebrae. Examples include the method described in [19], which fitted a central spinal curve using the center points of the spine. It trained a support vector machine to distinguish between two consecutive vertebrae, finding the plane between spinal regions of interest as the intervertebral disc plane. The Cobb angle was calculated based on the angles between these intervertebral disc planes. For patients with severe spinal deformities, where spinal morphology might be significantly distorted and compressed, relying solely on midpoints for curve fitting can result in a loss of information and inaccurate fitting. This research in general belongs to the “curve-based” methods, which generates a spinal curve but employed deep learning to generate a limited number of control points and knots, rather than spinal centers. This reduces the number of predicted points and can thus avoid overfitting of the curve. Besides, the dataset is this study exclusive focus on scoliosis cases ensures that the training is more specific and effective for this condition, which is practically important for surgical planning and intervention.

In this study, we demonstrated that automated 3D Cobb angle measurements from CT scans show a strong correlation with traditional 2D radiograph measurements. Despite the higher radiation exposure from CT scans, their use can be justified in certain clinical situation. CT imaging provides comprehensive 3D views of spinal deformities, which is particularly valuable in severe scoliosis cases. The ability to accurately assess vertebral rotations and alignments in three dimensions offers significant advantages for surgical planning and intervention. However, it is essential to balance the benefits of detailed anatomical information with the risks associated with increased radiation exposure. For routine scoliosis monitoring, traditional radiographs may suffice. In contrast, for preoperative evaluations and complex cases where detailed 3D visualization is critical, the use of CT scans can be justified. Clinicians should carefully consider the clinical context and prioritize imaging modalities based on the specific needs of each patient.

There are several limitations in this study. Firstly, the validation was performed based on limited internal samples, instead of large-scale external dataset. The main reason is the difficulty in acquiring public data for AIS patients with both CT data and X-ray. In the data selection process, researcher also encountered inconsistencies in the field of view of CT and X-Ray—for example, some X-rays covered the entire spine while the corresponding CT scans only covered a part, and in some cases, did not fully cover the scoliosis curvature. This discrepancy resulted in the exclusion of a subset of data from the experiment, which led to a relatively small sample size in this study. Secondly, the result (3D Cobb angle) only compared with the Cobb angle obtained from the 2D X-ray. However, the posture and imaging acquisition are different in CT and 2D X-ray. Even mapped the 3D spinal curve onto the coronal plane to get a MAP-2D-CA, but it is still a 3D Cobb angle. In the future, the further verification can be carried out by other 3D Cobb angle calculation methods. Thirdly, in this study, did not distinguish between different types or location of the scoliotic curve and did not conduct a subgroup analysis.

### Conclusion

This study presents a novel computational approach for measuring the three-dimensional Cobb angle from CT images in severe scoliosis patients. By leveraging 3D CT data, our method addresses the limitations of 2D radiographic measurements, providing a more accurate analysis of spinal deformities. The deep learning-based segmentation automates the process, reducing human error and increasing efficiency. High correlation with the clinical gold standard confirms the method’s clinical relevance. Our approach also handles severe scoliosis cases effectively, highlighting its robustness. Future studies will explore detailed correlations with clinical features to further understand the clinical value, incorporating a more diverse dataset and additional parameters to enhance its applicability. Overall, our method offers significant advancements in accuracy, efficiency, and clinical utility for preoperative planning and assessment in scoliosis patients. While CT imaging involves higher radiation exposure, its use should be reserved for cases where the detailed anatomical information significantly benefits clinical outcomes. Future research should focus on optimizing imaging protocols to minimize radiation exposure while maximizing diagnostic and surgical planning benefits.

## Data Availability

The data and code can be available by contacting the corresponding author at lynn2018.li@connect.polyu.hk.

## References

[1] Jada, A., C.E. Mackel, S.W. Hwang, A.F. Samdani, J.H. Stephen, J.T. Bennett, A.A. Baaj: Evaluation and management of adolescent idiopathic scoliosis: a review. Neurosurgical focus 43(4): E2–E2, 2017. 28965447 10.3171/2017.7.FOCUS17297

[2] Bachmann, K.R., E. Lu, W.M. Novicoff, P.O. Newton, M.F. Abel: The Lumbosacral Takeoff Angle Can Be Used to Predict the Postoperative Lumbar Cobb Angle Following Selective Thoracic Fusion in Patients with Adolescent Idiopathic Scoliosis. J Bone Joint Surg Am 102(2): 143–150, 2020. 31644521 10.2106/JBJS.19.00287

[3] Chen, I.H., C.W. Chen, M.H. Hu, P.Y. Wang, Y.C. Yeh, Y.F. Lee, P.L. Lai, and S.H. Yang: Simultaneous Hypercorrection of Lowest Instrumented Vertebral Tilt and Main Thoracic Curve is Associated With Progression of Residual Lumbar Curve in Adolescent Idiopathic Scoliosis. Spine 47(19): 1362–1371, 2022. 35867582 10.1097/BRS.0000000000004403

[4] Wan, S.H., D.L. Wong, S.C. To, N. Meng, T. Zhang, and J.P. Cheung: Patient and surgical predictors of 3D correction in posterior spinal fusion: a systematic review. Eur Spine J 32(6): 1927–1946, 2023. 37079078 10.1007/s00586-023-07708-2

[5] Bai, J., S. Liu, C. Liu, Y. Zhao, M. Li: Proximal junctional kyphosis in Lenke 5C adolescent idiopathic scoliosis after selective posterior thoracolumbar/lumbar fusion: risk factors and predictive index. J Orthop Surg Res 19(1): 24, 2024. 38167043 10.1186/s13018-023-04470-5 PMC10763114

[6] Shi, B., S. Mao, L. Xu, X. Sun, Z. Liu, J.C.Y. Cheng, Z. Zhu, Y. Qiu: Accurate prediction of height loss in adolescent idiopathic scoliosis: Cobb angle alone is insufficient. European Spine Journal 25(10): 3341–3346, 2016. 27001137 10.1007/s00586-016-4530-4

[7] van Popta, D., J. Stephenson, R. Verma: Change in spinal height following correction of adolescent idiopathic scoliosis. The Spine Journal 16(2): 199–203, 2016. 26515395 10.1016/j.spinee.2015.10.027

[8] Hwang, S.W., A.F. Samdani, B.S. Lonner, M.C. Marks, T.P. Bastrom, R.R. Betz, P.J. Cahill: A multicenter analysis of factors associated with change in height after adolescent idiopathic scoliosis deformity surgery in 447 patients. Journal of Neurosurgery: Spine 18(3): 298–302, 2013. 23330975 10.3171/2012.12.SPINE12870

[9] Kwan, M.K., C.K. Chiu, M.S. Hasan, S.H. Tan, L.H. Loh, K.S. Yeo, W.H. Lee C.Y.W. Chan: Perioperative Outcome of Single Stage Posterior Spinal Fusion for Severe Adolescent Idiopathic Scoliosis (AIS) (Cobb Angle ≥ 90°): The Role of a Dual Attending Surgeon Strategy. Spine 44(6): E348–E356, 2019. 30130336 10.1097/BRS.0000000000002848

[10] Mehta, N., B. Garg, T. Bansal, A. Aryal, N. Arora, V. Gupta: Predictors of Operative Duration in Posterior Spinal Fusion for Adolescent Idiopathic Scoliosis: A Retrospective Cohort Study. International Journal of Spine Surgery 16(3): 559–566, 2022. 35772986 10.14444/8251 PMC9650161

[11] Soliman, H.A.G., M. Beausejour, J. Joncas, M. Roy-Beaudry, S. Barchi, J.-M. Mac-Thiong, H. Labelle, G. Grimard, and S. Parent, Predicting lowest hemoglobin level and risk of blood transfusion in spinal fusion surgery for adolescent idiopathic scoliosis. European Spine Journal 28(6): 1342–1348, 2019. 30848365 10.1007/s00586-019-05939-w

[12] Yang, C., Y. Li, M. Yang, Y. Zhao, X. Zhu, M. Li, G. Liu: Adding-on Phenomenon After Surgery in Lenke Type 1, 2 Adolescent Idiopathic Scoliosis: Is it Predictable? Spine 41(8): 698–704, 2016. 26630420 10.1097/BRS.0000000000001303

[13] Schulz, J., J. Asghar, T. Bastrom, H. Shufflebarger, P.O. Newton, P. Sturm, R.R. Betz, A.F. Samdani, B. Yaszay, H.S. Group: Optimal Radiographical Criteria After Selective Thoracic Fusion for Patients With Adolescent Idiopathic Scoliosis With a C Lumbar Modifier: Does Adherence to Current Guidelines Predict Success? Spine 39(23): E1368–E1373, 2014. 25188601 10.1097/BRS.0000000000000580

[14] Nugent, M., R.C. Tarrant, J.M. Queally, P. Sheeran, D.P. Moore, P.J. Kiely: Influence of curve magnitude and other variables on operative time, blood loss and transfusion requirements in adolescent idiopathic scoliosis. Irish Journal of Medical Science 185(2): 513–520, 2016 25935207 10.1007/s11845-015-1306-5

[15] Yu, X., H. Xiao, R. Wang, Y. Huang: Prediction of Massive Blood Loss in Scoliosis Surgery From Preoperative Variables. Spine 38(4), 2013. 10.1097/BRS.0b013e31826c63cb 22872215

[16] Mimura, T., J. Takahashi, S. Ikegami, S. Kuraishi, M. Shimizu, T. Futatsugi, M. Uehara, H. Oba, M. Koseki, H. Kato: Can surgery for adolescent idiopathic scoliosis of less than 50 degrees of main thoracic curve achieve good results? Journal of Orthopaedic Science 23(1): 14–19, 2018. 28943143 10.1016/j.jos.2017.09.006

[17] Pasha, S., P.J. Cahill, J.P. Dormans, J.M. Flynn: Characterizing the differences between the 2D and 3D measurements of spine in adolescent idiopathic scoliosis. European spine journal 25(10): 3137–3145, 2016. 27146809 10.1007/s00586-016-4582-5

[18] Jin, C., S. Wang, G. Yang, E. Li, Z. Liang: A Review of the Methods on Cobb Angle Measurements for Spinal Curvature. Sensors (Basel) 22(9), 2022. 10.3390/s22093258 PMC9101880 35590951

[19] Huo, X., J.Q. Tan, J. Qian, L. Cheng, J.H. Jing, K. Shao, B.N. Li: An Integrative Framework for 3D Cobb Angle Measurement on CT Images. Computers in biology and medicine 82: 111–118, 2017. 28183004 10.1016/j.compbiomed.2017.01.007

[20] Alukaev, D., S. Kiselev, T. Mustafaev, A. Ainur, B. Ibragimov, T. Vrtovec: A deep learning framework for vertebral morphometry and Cobb angle measurement with external validation. European spine journal 31(8): 2115–2124, 2022. 35596800 10.1007/s00586-022-07245-4

[21] Wang, C., M. Ni, S. Tian, H. Ouyang, X. Liu, L. Fan, P. Dong, L. Jiang, N. Lang, H. Yuan: Deep learning model for measuring the sagittal Cobb angle on cervical spine computed tomography. BMC Med Imaging 23(1): 196, 2023. 38017414 10.1186/s12880-023-01156-6 PMC10685593

[22] Lessmann, N., B. van Ginneken, P.A. de Jong, I. Išgum: Iterative fully convolutional neural networks for automatic vertebra segmentation and identification. Medical image analysis 53: 142–155, 2019. 30771712 10.1016/j.media.2019.02.005

[23] Qadri, S.F., H. Lin, L. Shen, M. Ahmad, S. Qadri, S. Khan, M. Khan, S.S. Zareen, M.A. Akbar, M.B. Bin Heyat, and S. Qamar, CT-Based Automatic Spine Segmentation Using Patch-Based Deep Learning. International Journal of Intelligent Systems 2345835, 2023.

[24] Altini, N., G. De Giosa, N. Fragasso, C. Coscia, E. Sibilano, B. Prencipe, S.M. Hussain, A. Brunetti, D. Buongiorno, A. Guerriero, I.S. Tatò, G. Brunetti, V. Triggiani, V. Bevilacqua: Segmentation and Identification of Vertebrae in CT Scans Using CNN, k-Means Clustering and k-NN. Informatics (Basel) 8(2): 40, 2021.

[25] Liang, Z., Q. Wang, C. Xia, Z. Chen, M. Xu, G. Liang, Z. Yu, C. Ye, Y. Zhang, X. Yu, H. Wang, H. Zheng, J. Du, Z. Li, J. Tang: From 2D to 3D: Automatic measurement of the Cobb angle in adolescent idiopathic scoliosis with the weight-bearing 3D imaging. Spine J. 10.1016/j.spinee.2024.03.019, 2024. 38583576 10.1016/j.spinee.2024.03.019

